# Anti-thrombotic trials in atrial fibrillation, the RELY study

**Published:** 2010

‘The strength of the RELY study of dabigatran in stroke prevention resides in two critical factors; (1) the scientific integrity of the study despite being unblinded to warfarin, and (2) the wide range of patients at risk for stroke studied.’

This view was expressed by the co-principal investigator of the study, Prof Michael Ezekowitz in an interview with the Cardiovascular Journal of Africa at the 2010 South African Heart Congress in August at Sun City.

Dr Ezekowitz graduated from the University of Cape Town Medical School. After residency he proceeded to London University (Imperial College) on a British Council fellowship where he was awarded a Doctor of Philosophy degree. His fellowship in cardiology was at Johns Hopkins Hospital. He has held faculty appointments at the University of Oklahoma and Yale University School of Medicine.

In 2000 he moved to Philadelphia as the June F Klinghoffer professor and chairman of Medicine at Drexel University School of Medicine. He is now the vice president of Lankenau Institute for Medical Research (LIMR) and vice president of Clinical Research, Main Line Hospitals, and professor at Jefferson Medical College.

‘In retrospect, RELY was an amazing study; we were surprised by the results, on the upside!’ RELY was primarily designed to determine the safety and efficacy of dabigatran in patients with atrial fibrillation at risk of stroke, compared to the gold standard, warfarin. Warfarin had already been shown to be superior to aspirin and clopidogrel for stroke prevention in the ACTIVE-W trial.^2^

‘In RELY, the higher dose of dabigatran, 150 mg bid, was shown to be significantly and statistically superior to warfarin with regard to its efficacy in stroke prevention; while the lower dose (110 mg bid) equalled warfarin efficacy but was significantly safer than warfarin’, Dr Ezekowitz pointed out.

There was no price to pay with regard to bleeding rates and surprisingly there was between 60 and 70% reduction in intracranial haemorrhage in the dabigatran-treated groups. A dose-related response was seen in the two doses of dabigatran used for efficacy and safety.

‘We had hoped to fast track the registration of this indication for dabigatran as it has the potential to change clinical practice radically with regard to atrial fibrillation. However, the FDA, as it should, is subjecting the data to rigorous evaluation. I am personally confident that we will get approval.’

Results of the RELY study were documented in a number of presentations at the 2009 ESC congress in Barcelona to an audience of 8 000 to 10 000 cardiologists from around the world. Criticisms of the study have been few, mainly directed at the single-blinded warfarin arm and the issue of differences in myocardial infarctions, which were slightly higher in the dabigatran group.

Dr Ezekowitz commented that the design of the study was very carefully considered, ‘The dabigatran dosage arms were double blind, but the warfarin arm was not. We made very important safeguards to maintain the scientific integrity of the trial – all hospital admissions were assessed blindly for both safety and efficacy end-points. Questionnaires were completed by every patient at every visit to ensure that minor events and sideeffects were not missed.’

The increase in clinically manifested myocardial infarctions (MI) of 0.2% per year in the dabigatran arm compared to the warfarin arm (with no difference in fatal MI) was looked at more closely after the announcement of the results. ‘Myocardial infarction rates were very low throughout the study. When silent ischaemia was included, there was no statistical difference between the warfarin and dabigatran-treated patients’, Dr Ezekowitz noted.

Importantly, there was no evidence of liver toxicity. This was one of the most meticulously conducted aspects of the RELY study, following the failure of an earlier direct thrombin inhibitor, xymelagatran, as a result of liver toxicity. ‘I can state unequivocally that this drug is not toxic to the liver’, Dr Ezekowitz stressed.

Of importance is that patients at lower risk of stroke, at a CHAD score of 1, showed a striking benefit in stroke reduction when treated with dabigatran compared to warfarin. In patients (about 2 000) who underwent cardioversion, overall complication rates were very low and comparable to those on warfarin, which should increase confidence in using this effective strategy when patients are on dabigatran.

There are limitations to the use of dabigatran. It cannot be used at creatinine clearance levels < 30 ml/min and some patients are not able to tolerate the dyspepsia. In the RELY study, 2.5% of patients withdrew from the dabigatran arm due to this side effect.

There are some drug interactions with dabigatran, for example proton pump inhibitors reduce absorption, but the only drug truly contra-indicated for patients on dabigatran is quinidine.

Dabigatran has shown that three out of four potential strokes caused by atrial fibrillation can be prevented. Atrial fibrillation patients eagerly await this drug.
